# Supplementary material for: Causal Relationship between Aging and Anorexia Nervosa: A White-Matter-Microstructure-Mediated Mendelian Randomization Analysis
Source: Biomedicines. 2024 Aug 16;12(8):1874. doi: 10.3390/biomedicines12081874 (PMC11351342; doi:10.3390/biomedicines12081874)
Supplement: Supplementary file 1 [file biomedicines-12-01874-s001.zip › Supplementary Figure/Figure S8/Leaveoneout plot of MR analysis for TL to ubm-b-1617.pdf]

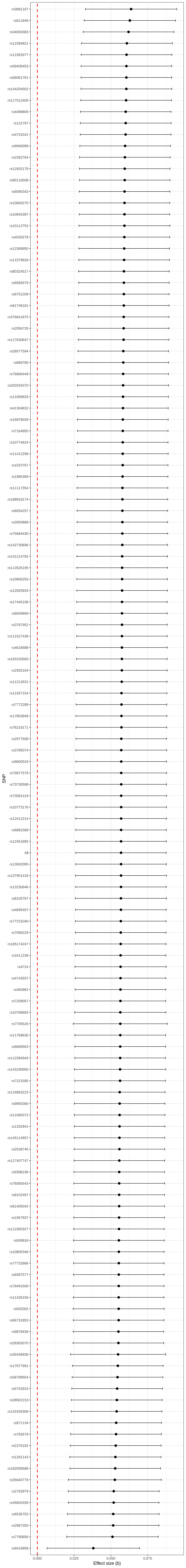
rs3891167
rs611646
rs34550383
rs11584821
rs11991877
rs59409453
rs56061761
rs144204502
rs117512405
rs4498805
rs131797
rs4731541
rs9940099
rs2282764
rs12932179
rs80116508
rs6590343
rs10840270
rs10845387
rs10112752
rs4530278
rs12369950
rs11579626
rs80324517
rs6584579
rs6751209
rs61748181
rs376641875
rs2056726
rs117630647
rs28577594
rs869785
rs76666449
rs202034370
rs11699029
rs41304832
rs16978028
rs7164950
rs10774624
rs11412296
rs1023767
rs1985369
rs11117354
rs188918174
rs6054257
rs3093888
rs75664430
rs142730696
rs141214782
rs113525195
rs10905255
rs12925933
rs17445108
rs6659669
rs3767952
rs111527438
rs4616688
rs150150565
rs2555104
rs11212631
rs11557154
rs7772289
rs17803849
rs76219171
rs2977608
rs3785074
rs9600019
rs79977579
rs73730598
rs73581419
rs10773176
rs12412214
rs6881568
rs12451892
A8
rs13062095
rs137901416
rs13230646
rs8105767
rs4695407
rs77231040
rs7099229
rs185174247
rs1611236
rs4724
rs4743037
rs450962
rs7209057
rs10768683
rs7705526
rs11769630
rs6669563
rs112394943
rs143190905
rs7221585
rs116863223
rs9955360
rs11085072
rs1332941
rs145114957
rs2538745
rs117407147
rs9398196
rs76065543
rs8102497
rs61405042
rs1957937
rs111950327
rs939916
rs10805346
rs77732866
rs6587577
rs78491606
rs11426156
rs932002
rs66731853
rs9878436
rs28363070
rs35446936
rs17677991
rs56799554
rs5742915
rs28502153
rs142426306
rs871134
rs762679
rs2276182
rs1291143
rs182059586
rs35640778
rs2763979
rs45604339
rs6536702
rs2967355
rs7790856
rs9419958
SNP
0.000
0.025
0.050
0.075
Effect size (b)
